# Monthly Summary

**Published:** 1896-05

**Authors:** 


### Monthly Summary.

DENTISTRY IN BRAZIL.—The climate, an all-important factor to the foreigner, is, of course, distinctly tropical, accompanied by that lavish display of vegetation characteristic of the tropics—becoming in the lowlands a perfect net-work of palms, mosses, grasses, etc., luxuriant beyond description. The summer season, from about December 1st to April, is extremely hot, and as the large cities are all seaport towns

(with the exception of Sao Paulo), low and damp, they become veritable incubators for low fever germs which, to the unacclimated new-comer, are often fatal, but in other seasons it is often pleasant indeed, and, till the novelty wears off, is really quite ideal.

The people, a mixture of the Portuguese, African negro and native Indian, are an olive complexioned, rather slight people—the better class are better to meet and quite cordial, but inclined to be hypercritical. Their government is at present a very unstable affair, which is detrimental to money exchange and cripples the country. For admittance to practice dentistry in your own name, an examination in the Portuguese language before the Medical Board of Rio de Janeiro must be taken, which, by the way, is very severe, but by being in the employ of or using the name of some one licensed, you are not interfered with.

Practically all of the representative dentists are North Americans, who employ from three to five more, but there are some native practitioners who come to "the States" for their course, then return. Prices being high, the work among these representatives is of a very superior quality and conscientiously done. Their offices are fully equipped with all the late appliances; but there are native dentists who are about as numerous as barbers, and who with few exceptions, maintain about the same degree of dignity. Their outfit would include little more than forceps, amalgam, a spatula and arsenic. They use the latter about as freely as we would dental plaster—from an obtundant in a simple cavity to an exposure. The pulp is never removed, so when pulpitis or an abscess develops it is extracted, or they "save up their money" and apply to a "dentista Americana," whose appointment book usually has a dozen or more cases bearing evidence of the skill (?) of these native operators. Outside of the office the life is hardly as agreeable as most of us would wish, as for the first year you are handicapped by the language, and after you have mastered that after a fashion and feel that you would like to become acquainted and mingle somewhat in the society that is afforded, you are confronted with the fact that, humiliating as it may seem, a dentist holds an inferior position. This also holds good among the British residents. But grant that you gain the dental *entree*, you soon find their ways and what is expected of you so different from what you are accustomed to, that you are pleased to let it alone and return to your dental friends once more, where, with the exception of a dinner or an excursion "up country" now and then, you experience the steady roatine of work, eat and sleep.—*Dental Register.*

---

A MECHANICAL HORROR.—*Machinery* is a monthly journal published at Johannesberg, South Africa. In the October number, just received, is an account of a most remarkable clock belonging to a Hindu prince, which the editor thinks the strangest piece of machinery in India. Near the dial of an ordinary looking clock is a large gong hung on poles, while underneath, stattered on the ground, is a pile of artificial human skulls, ribs, legs and arms, the whole number of bones in the pile being equal to the number of bones in twelve human skeletons. When the hands of the clock indicate the hour of one, the number of bones needed to form a complete human skeleton come together with a snap, by some mechanical contrivance the skeleton springs up, seizes a mallet, and walking up the gong, strikes one blow. This finished, it returns to the pile and again falls to pieces. When two o'clock, two skeletons get up, and strike, while at the hours of noon and midnight the entire heap springs up in the shape of twelve skeletons, and strikes, each one after the other a blow on the gong, and then fall to pieces, as before.—*Items of Interest.*

---

SAVING TEETH.—A young lady of about twenty-one years came to me some weeks since for consultation. On examination, I found the following conditions: Left upper lateral incisor extracted, left central pulpless, right central with two very large cavities and pulp nearly exposed, right lateral broken off half way from gum to cutting edge, and the remaining teeth, though slightly irregular were of good form and structure, with only a few small crown cavities.

Gums fine and healthy, and general health excellent.

She had been told by another dentist that the three remaining incisors could not be saved, and that as it was only a question of time when the others would ache too, she must have them extracted.

Having known me in previous years, and wishing to save her teeth, if possible, she came across the State to me.

I devitalized the right central and lateral, treated and filled the roots of all three, and ground them down to the margin of the gum. To the left central I fitted a collar crown, to which I soldered the left lateral. The right central and lateral were fitted with porcelain-faced browns.

In the remaining teeth were placed six gold and two amalgam fillings, all small, and the teeth thoroughly cleansed and polished.

Result: Teeth preserved, facial contour retained, usefulness restored, appearance improved, and patient gratified.—Dr. H. B. Smellie, in *Items of Interest.*

---

TO STERILIZE STEEL INSTRUMENTS WITHOUT REDUCING THEIR TEMPER.—The usual process of sterilizing surgical instruments by pouring boiling water on them and then cooling gradually, removes their fine, high temper, rendering them nearly useless. Dr. Nathan Hermann, Louisville, in the "Louisville Medical Monthly," suggests the following improved method:

"A knife made of the best tempered steel and upon which all the cutler's care has been lavished to the end of supplying it with a perfect edge, will lose the same after having passed two or three times (varying with its original quality) through this sterilizing ordeal, nor will it ever afterward be capable of holding an edge.

"The process of sterilization which I propose to substitute, and I have already used it myself with excellent results, is to cover the instruments as before with boiling water, but to leave them in their bath but for one minute, when the water is to be poured off or drained off with a siphon, after which they are to be covered with iced lime-water. The temper of the steel is thus preserved or even improved, while the principle of asepsis is also carried out.

"Cold lime-water is the best known means of hardening steel after its semperature has been raised; and the small quantities of lime water adhering to the instrument can not harm the most delicate tissues. In delicate operations, as in laparotomies, the surgeon can prepare his own lime-water by pouring distilled water over lime, when the elevation of temperature incidental to the mixture of lime and water will preclude the possibility of the survival of the germ. The vessel is then closed with a sterilized cotton plug, and set aside to await the pleasure of the surgeon. Some minutes before the operation, a sufficient quantity of this lime-water is decanted into a sterilized vessel containing artificial ice, which, as is well known, is made of distilled water, and you have a thoroughly sterile and innocuous tempering fluid."

---

THE dentist must be physically sound, without health he will be unable to bring the serviceable attributes and the fertile brain so necessary in the successful practice of his profession.

His manner should be good; his demeanor kind and gentle, indicating the inner worth.

He must be morally sound. This is the keynote to the symphony, the compass of the ship, the main path to the broad highway of success. The moral safety of the patients entrusted to his care must be an assurance beyond peradventure; like Cæsar's wife he must be above suspicion.

He must collect his bills and pay his debts.—C. E. Bentley, in *Items of Interest.*

---

RELIEF OF HEADACHE FOLLOWING DENTAL OPERATIONS.—One drop of nitro-glycerin (one per cent solution) in half a glass of water will give prompt relief in many cases.—E. H. Browne, in *Items of Interest.*

ADVANTAGES OF PYROZONE.—Pyrozone, in the three per cent. solution, is rapidly gaining favor with surgeons in hospitals where the benefits of a non-poisonous solution of $H_2O_2$ can be readily shown by comparison with solutions containing barium and other injurious foreign substances.

Very great improvements have lately been made in the manner of putting up solutions of pyrozone, so that decomposition of the peroxid of hydrogen is now avoided.

Pyrozone three per cent. solution may be ordered in the new amber glass stoppered bottles, and it will keep for an indefinite period. The constantly increasing use of pyrozone is demonstrating that its use is closely in accord with the most recent germ theories of disease.

It is used with excellent effect at the toilet table, as well as in the surgery, and has the indorsement of those who give zymotic reasons for the spread of disease.

In private families where there is invalidism, with its unpleasant odors and its dangerous excretions, pyrozone three per cent. solution should be liberally used as a deodorant, pus destroyer and general germicide. It is harmless to living tissue, and can be taken internally with benefit in many troubles affecting the stomach.—*The National Board of Health Magazine.*

---

BALSAM VARNISH.—Dr. Howard's Antiseptic Varnish for coating cavities preparatory to filling, consists of Canada balsam, to which has been added mercuric chloride and thymol, evaporated over a water bath from twenty to twenty-eight hours, and finally dissolved in chloroform. The proper consistency can only be determined by experience and careful observation, and it is upon this that its usefulness depends. When improperly prepared it is valueless. It is not, of course, intended to retain fillings, but to aid in their adaptation and to act as an anti-thermal and protective coating. This formula has been annually given to the classes of Dr. Howard in the University of Buffalo.—*Dental Practitioner and Advertiser.*

FILLING PULPLESS TEETH WITH FISTULOUS OPENING.—In the May number of the "Items of Interest," Dr. C. N. Johnson, of Chicago, tells how he does this operation. The busy practitioner may not have time to resort to this proceeding, as described by Dr. Johnson. Now let me tell you how to do the operation, and do it quickly.

After getting a direct opening into the pulp chamber, and thoroughly washing out its contents with warm water from a syringe, apply the rubber-dam, and dry the pulp chamber with cotton and hot air, then with Gates-Glidden drills of different sizes enlarge the canals to the apex; now twist a few shreds of cotton around a Donaldson broach, and saturating in pure carbolic acid and iodoform, use as a piston till the medicament appears at the fistulous opening, and does not cease till it does appear.

Now your tooth is ready to fill; do not wait a day or a week, but go right ahead and fill solidly to the apex. I use shreds of cotton saturated with chloro-percha, and a touch of iodoform, and have yet to see the first case of this kind come back to me for treatment in a practice of fifteen years.—Dr. August Cameron, in *Dominion Dental Journal.*

---

SPOT SPECIALISM.—This is a new term used to designate the specialist crank who goes on dividing a specialty into its subdivisions until only one spot is considered worthy of study. The term is commended to some of our friends who dislike the hard work of some departments of dental practice and desire to confine themselves to that which is easy and remunerative.—*Western Dental Journal.*

---

ADMINISTERING NITROUS OXID.—Especially in English dental journals do we see directions as to the use of the "gas bag," Gas should always be given from a gasomoter arranged so that the volume of gas comes freely and with even pressure. If it is given from the bag, the pressure varies greatly and the effort in breathing from the partially collapsed bag enhances the danger.—*Western Journal.*

PINK BASE PLATE.—In nearly every sense I know of nothing so important as pink base-plate gutta-percha.

To teach you how I use it for the preservation of the human teeth, both temporary and permanent, will be the foundation of a system that, if I have had any success at all, I can attribute to this one article as much or more than any other filling material.

In conjunction with this I cannot overestimate the value of this discovery of the laws of articulation and the articulator that bears my name, and of which I am more proud than of all my other productions. Without knowing what I do of articulation, the gutta-percha might never have been seen in the same light by me.—J. Foster Flagg, in *Items of Interest.*

---

SPECIFIC REMEDIES.—We are all familiar with the story of the botanical physician who used but three remedies, made from infusions of the bark of the same tree. If it were peeled upward it operated as an emetic, and the infusion was called hiloborum. If it were peeled downward, it operated as a cathartic, and became lobohirum. But when the bark was taken off around the tree, the infusion became that virulent poison hilobustem. This sounds a little absurd, but there are a great many dentists who, if we may believe their utterances in dental meetings and journals, rely almost exclusively upon their own system of hiloborums, lobohirums, and hilobustems. —*Dental Practitioner and Advertiser.*

---

In filling temporarily a cavity with an exposed, or nearly exposed pulp, gutta-percha is one of the worst substances to be used. Mastication is almost sure to press it sufficiently on to the pulp as to produce pain and perhaps inflammation, if not suppuration and death. Even the swelling of this material is sometimes fatal. After you have removed as much of the decay as you can, without disturbing the pulp, and you have placed on it a very little chloroform, tinctured with equal parts of oil of cloves and carbolic acid, cover the tender portion with a slight coating of chloro-perch on a disc of paper, and then fill the cavity with oxiphosphate.

